# Differential proteomic of plasma provides a new perspective on scientific diagnosis and drug screening for dampness heat diarrhea in calves

**DOI:** 10.3389/fvets.2022.986329

**Published:** 2022-09-20

**Authors:** Zunxiang Yan, Kang Zhang, Guibo Wang, Lei Wang, Jingyan Zhang, Zhengying Qiu, Zhiting Guo, Kai Zhang, Jianxi Li

**Affiliations:** Engineering and Technology Research Center of Traditional Chinese Veterinary Medicine of Gansu Province, Lanzhou Institute of Husbandry and Pharmaceutical Sciences, Chinese Academy of Agricultural Sciences, Lanzhou, China

**Keywords:** biomarkers, calf, dampness heat diarrhea, parallel reaction monitoring, proteomic

## Abstract

Dampness heat diarrhea (DHD) is one of the most common syndromes of calf diarrhea. Its complex etiology and lack of objective diagnostic criteria bring great challenges to the diagnosis and treatment of this disease. This study aims to screen some prospective diagnostic biomarkers or therapeutic targets for calves with DHD by investigating the differential protein profiles of plasma between DHD calves and clinically healthy calves by mass spectrometry-based proteomic. A total of 120 DHD calves and 90 clinically healthy calves were divided into two groups randomly, 30 DHD calves and 30 clinically healthy calves in the test group, and 90 DHD calves and 60 clinically healthy calves in the validation group. In the test group, a total of 52 proteins were differentially expressed between calves with DHD and clinically healthy calves, 13 proteins were significantly increased and 39 proteins were significantly decreased. The differentially expressed proteins were associated with the intestinal immune network of IgA production, caffeine metabolism, purine metabolism, and PI3K signaling pathway. In the validation group, 13 proteins were selected from 52 differential expression proteins for parallel reaction monitoring validation to verify their associations with DHD calves. The targeted proteomic results showed that fibronectin precursor (FN1) and apolipoprotein C-IV precursor (APOC4) were significantly associated with DHD in calves, and they were downregulated in sick calves. In conclusion, the differential expression of plasma proteins was associated with DHD pathogenesis in calves, and the FN1 and APOC4 might be the potential clinical biomarkers for diagnosis of DHD in calves, and the intestinal immune network of IgA production, caffeine metabolism, purine metabolism, and PI3K signaling pathway are the candidate targets to treat DHD in calves. Our finding provides a reference for further investigating the pathogenesis, developing techniques of diagnosis, and screening treatment drugs for DHD in calves.

## Introduction

Diarrhea is one of the most common diseases worldwide in the cattle industry and can be caused by various complicated factors (1, 2). Especially, the morbidity and mortality of neonatal calf with diarrhea are high, which imposes a heavy economic burden on the cattle industry. According to the theory of traditional Chinese veterinary medicine (TCVM), diarrhea can be divided into dampness-heat diarrhea (DHD), dampness-cold diarrhea, spleen deficiency diarrhea, and kidney deficiency diarrhea (3–5). DHD is one of the most common syndromes in Chinese cattle herds, and its main clinical symptoms in calves are hyperthermia, sticky and loose stools with blood, mucus or purulence, red tongue, and thick greasy tongue-coating (3, 6). Although the management, feeding, nutrition, and equipment of cattle farms have been greatly improved, the DHD in calves is difficult to be effectively controlled in clinical practice due to the complicated etiology. Therefore, it is of great clinical significance to study the pathogenesis of calves with DHD from the whole perspective and to find suitable biomarkers for the diagnosis and targets for screening treatment drugs for this disease. However, there is a lack of systematic studies on the pathogenesis of DHD in calves, especially, since there are few of published evidence at the proteomics level. As an important method for screening biomarkers on various diseases, systems biology can help to find suitable and less invasive markers for diagnosis and targets for the treatment of calves with DHD.

With the rapid development of multi-omics analytical techniques, they have been widely used to explore the mechanism of various diseases, including gastrointestinal diseases (7, 8), cardiovascular diseases (9), and cancers (10). Few research on multi-omics techniques focused on calf diarrhea. Recently, metabolomics was used to study the mechanism of diarrhea in calves, and acetylcarnitine, indoxyl sulfate, and oxindole have been recognized to be potential biomarkers for calf diarrhea (11). Additionally, metabolites such as lecithin and choline have been found to affect the progression of DHD by interfering with the metabolism of arachidonic acid, linoleic acid, and glycerophospholipids in calves (12). Previous studies on the structure and function of fecal microflora of calves with DHD indicated that DHD had a significant effect on the intestinal microbial compositions in calves, including significant changes in Escherichia-Shigella, Bacteroides, and Fournierella in sick calves (12). These studies imply that systems biology makes it possible to screen potential pathological targets for calves with DHD.

At present, because there are few studies on the pathogenesis of calves with DHD, veterinarians can only confirm the diagnosis of this disease in calves by clinical manifestations. Biomarkers are objective measurements of biological processes, which can help diagnose DHD in calves more accurately and understand the mechanism of the disease. Therefore, systems biology is used to study calves' DHD, which might offer new practical information for the improvement of treatment strategies and diagnostics. However, there is no report about the systematic study on calves' DHD from the perspective of differential proteins. Blood samples in calves are easy to be obtained and have abundant proteins, which make them more suitable for clinical detection. The data-independent acquisition (DIA) is a high-throughput, high-sensitivity, and accurate approach for the identification of biomarkers in various diseases (13, 14).

This study aimed to identify potential biomarkers from plasma protein associated with DHD of calves. The experiment was conducted in two groups. In the test group, proteomic analysis of plasma was performed using DIA-mass spectrometry, the aim was to discover the proteins with significant changes in calves with DHD. In the validation group, parallel reaction monitoring (PRM) technology was used to validate the differential proteins for improving the reliability of these proteins as markers on calves with DHD. As a result, there were 52 proteins differentially expressed between calves with DHD and clinically healthy calves, 13 proteins were significantly increased and 39 proteins were significantly decreased. FN1 and APOC4 were significantly associated with DHD in calves. The differentially expressed proteins were related to the intestinal immune network of IgA production, caffeine metabolism, purine metabolism, and PI3K signaling pathway.

## Materials and methods

### Animal recruitment

The trial was carried out on a commercial dairy farm in Hui Autonomous Prefecture of Changji, Xinjiang Uygur Autonomous Region, northwest of China. The breed of the calf is Holstein, and all enrolled calves were similar in genetic background and age (10- to 20-day old), and housed individually in a calf shed under the same conditions. The calves were fed colostrum at a dose of 4 kg/calf only on the first day of postpartum, and then fed the pasteurized normal milk at a dose of 6 kg/day, and the calf feed was fed from the third day of postpartum, and the feeding and drink tools were washed and disinfected two times daily. The diagnosis of calves with DHD followed the protocol in our previous study (12), which means that the DHD case was mainly characteristic with diarrhea as well as the symptom of red tongue coated with thick greasy tongue-fur or the symptom of feces with mucus, blood, or purulence. At the same time, the DHD case must show two out of total minor symptoms in Table 1. The inclusion and exclusion criteria of DHD cases are presented in Table 2. All of the clinically healthy calves showed normal body temperature without any abnormal clinical manifestations. The inclusion and exclusion criteria for the clinically health cases are presented in Table 3. The specific clinical syndrome differentiation was carried out by a senior expert and a Ph.D. student, their major is traditional Chinese veterinary medicine and clinical diagnosis. The screening protocol of DHD cases and clinically healthy calves were performed according to veterinary clinical examination such as the diagnosis protocol of DHD and the inclusion and exclusion criteria. The test calves were recruited from early of June to the end of July 2020. The enrolled calves were separated into a test group and a validation group. The test group consisted of 30 calves with DHD and 30 clinically healthy calves. The validation group consisted of 90 calves with DHD and 60 clinically healthy calves. The study protocols were approved by the guidelines of the Laboratory Animal Ethics Commission of the Lanzhou Institute of Husbandry and Pharmaceutical Sciences of CAAS (SYXK [Gan] 2019-0002).

**Table 1:** Diagnostic criteria for DHD calves.

**Symptoms**	**Clinical signs**
Major symptoms	1. Diarrhea
	2. Mucus or bloody or purulent stool
	3. Red tongue and thick greasy tongue-fur
Minor symptoms	1. Hyperthermia, dry nose
	2. Abdominal pain, tenesmus
	3. Anal red and swell
	4. Thirst and small amount, shortness of urination

Diarrhea, calf excreted semiliquid or watery feces. Mucus or bloody or purulent stool, mucus, blood, or purulence was observed in feces. Red tongue, the color of tongue in sick calf was redder than the normal color (reddish) of tongue in the healthy calf. Thick greasy tongue-coating, the surface of the tongue was covered fully or partly by a yellow thick tongue fur. Hyperthermia means high body temperature. Shortness of urination, the volume of urine was reduced in sick calves. Abdominal pain, the intestine or colon of sick calf showed the feeling of pain. Anal was red and swell. Loose stools like water, much of water was in feces. Tenesmus, sick animal excreted frequently feces, but the volume of feces was obviously reduced per time. Dry nose, the tip of nose was dry. Thirst and small amount, the desire to drink water was strong, but the intake volume of water was less.

**Table 2:** Inclusion and exclusion criteria for DHD calves.

**Inclusion criteria**	**Exclusion criteria**
Compatible with the diagnostic protocol	With pneumonia or other clinical diseases
Age <3 weeks old	Age more than 3 weeks old
Untreated with antibiotics or other drugs	Treated with antibiotics or other drugs

**Table 3:** Inclusion and exclusion criteria for clinically healthy calves.

**Indexes**	**Inclusion criteria**	**Exclusion criteria**
Mobility	Actively mobile	Slight depression
Body temperature	Normal	Over 39°C
Appetite	Good	Slightly poor
Stool form	Formed stool	Unformed and abnormal stool
Age	Less than 3-week-old	More than 3-week-old

### Plasma sample collection

Blood samples were drawn from the jugular vein and collected in tubes containing ethylene diamine tetraacetic acid (EDTA). Approximately 10 ml of venous blood was centrifuged at 5,000 g for 15 min at 4°C to obtain plasma. After all plasma samples of enrolled animals were collected, 30 plasma samples of DHD cases and 30 plasma samples of clinically healthy calves were divided into the test group, and 90 plasma samples of DHD cases and 60 plasma samples of clinically healthy calves were divided into the validation group. Before protein extraction, every 10 individual similar samples with equal volume were mixed to generate one pooled sample. Therefore, 3 DHD and 3 healthy pooled samples were, respectively, subjected to protein extraction for proteomic analysis in the test group, and 9 DHD and 6 healthy pooled samples were, respectively, subjected to protein extraction for proteomic analysis in the validation group. The plasma samples were immediately stored in liquid nitrogen until further protein extraction.

### Protein sample preparation

After the pooled plasma samples were thawed at 4°C, which were transferred to a new centrifuge tube containing lysis buffer (2% SDS, 7 M urea, 1 mg/ml protease inhibitor cocktail) and homogenized by an ultrasonic homogenizer (PS-60AL, Leidebang Electronics Co., LTD, Shenzhen, China), proteins were isolated by centrifugation at 21,000 g for 15 min at 4°C (Eppendorf 5427R, Eppendorf, German) and their concentrations were measured using the BCA protein assay kit (Beyotime Biotechnology Co., LTD, Shanghai, China).

A total of 50 μg protein of each pooled sample was suspended in 50 μl deionized water and incubated at 55°C for 1 h after adding 1 μl 1 M dithiotreitol (DTT), then added 5 μl 20 mM iodoacetamide and alkylated at 37°C for 1 h in dark. Subsequently, 300 μl cold acetone was added to precipitate protein at −20°C overnight. The precipitate was washed twice with prechilled acetone and then resuspended in 50 mM ammonium bicarbonate. Finally, the protein was digested with sequence-grade modified trypsin (Promega, Madison, WI) according to a substrate/enzyme ratio of 50:1 (w/w) at 37°C for 16 h.

### High-performance liquid chromatography-tandem mass spectrometry (HPLC-MS/MS) analysis

The pooled sample was re-dissolved in 30 μl solvent A (A: 0.1 % formic acid in water) and analyzed by online nanospray LC-MS/MS on an Orbitrap Fusion Lumos coupled to EASY-nLC 1200 system (Thermo Fisher Scientific, MA, USA). Three microliters plasma peptides were loaded onto the nano column (Acclaim PepMap C18, 75 μm × 25 cm) with a 120-min gradient, from 5 to 35% in solvent B (B: 0.1% formic acid in ACN). The column flow rate was maintained at 200 nl/min with a column temperature of 40°C. The electrospray voltage of 2 kV vs. the inlet of the mass spectrometer was used. The mass spectrometer was run under data independent acquisition mode and automatically switched between MS and MS/MS mode. Three replicates were performed for pooled samples in the protein identification. The specific parameters are in the Supplementary material.

### Protein functional annotation and enrichment analysis

The biological significance and ontological functions of the differential proteins were analyzed using Gene Ontology (GO) annotation. The pathway of the proteins was annotated using the Kyoto Encyclopedia of Genes and Genomes (KEGG). Pathway-based analysis was used to further understand how different proteins coordinate each other to perform their biological functions. Significant ontological functions and pathways were examined within differentially expressed proteins with *P* < 0.05. We used the interaction relationships in the STRING protein interaction database (http://string-db.org) to analyze the differential protein interaction network and construct the protein interaction network diagram (15).

### Database search and data analysis

Raw Data of DIA were processed and analyzed by Spectronaut X (Biognosys AG, Switzerland) with default settings to generate an initial target list. Spectronaut was set up to search the database of cattle along with the contaminant database assuming trypsin as the digestion enzyme. Carbamidomethyl was specified as the fixed modification for cysteine. Oxidation was specified as the variable modification for methionine. Comparisons between the DHD group and healthy group were performed using Student's *t*-test. *Q*-value cutoff on precursor and protein level was applied at 1%. Proteins with significant differences between groups must meet the absolute value of the fold change (FC) >1.5 times, and the Q value obtained by correcting the *P*-value should be <0.05. The R packages “ggpubr” and “ggthemes” were applied to construct a volcano plot of the different proteins between the DHD and clinically healthy calves.

### Parallel reaction monitoring (PRM) and data analysis

To verify the reliability of the differential plasma proteins as diagnostic biomarkers for calf diarrhea, the expression levels of 13 proteins were further quantified by LC-PRM/MS analysis. These proteins were screened according to the unique peptides detected by mass spectrometry (>1), mass charge ratio, and the stability of retention. A total of 38 unique peptides were detected from these 13 differential proteins, which can be used for PRM. The PRM analysis was performed by Applied Protein Technology (Shanghai, China). The plasma proteins were isolated by centrifugation at 21,000 g for 15 min at 4°C, and their concentrations were measured using the BCA protein assay kit (Beyotime Biotechnology Co., LTD, Shanghai, China). Two hundred micrograms of protein of each sample was added to DTT at a final concentration of 100 mM. The samples were boiled for 15 min and cooled at room temperature. Then, each sample was mixed with 200 μl UA buffer (8 M urea, 150 mM Tris-HCl, pH 8.0) and centrifugated in an ultrafiltration tube at 14,000 g for 40 min. Discard the filtrate and add 100 μl IAA buffer [50 mM IAA (Bio-Rad, 163-2109) in UA] to shake at 150 g for 1 min. After incubation at room temperature for 30 min, each sample was centrifuged at 14,000 g for 20 min. Subsequently, each sample was washed with 100 μl UA buffer three times and with 100 μl NH_4_CO_3_ buffer (50 mM) for two times, and centrifuged at 14,000 g for 20 min. After adding 40 μl NH_4_CO_3_ buffer containing trypsin (1:50; Promega, 317107), the samples were shaken at 150 g for 1 min and incubated for 16 h at 37°C. Centrifugation at 14,000 g for 20 min and another 30 min following adding 40 μl NH_4_HCO_3_ buffer was used to collect the peptides. After desalting and lyophilization, the peptides were redissolved in buffer A (0.1% formic acid in water). The peptide content was estimated by UV light spectral density at 280 nm. Tryptic peptides were loaded onto a trap column (100 μm × 50 mm, 5 μm-C1) connected to a home-made tip column (75 μm × 200 mm, 3 μm-C18) for desalting before reversed-phase chromatography on an Easy nLC-1200 system (Thermo Fisher Scientific, MA, USA). One-hour liquid chromatography gradients with acetonitrile ranging from 5 to 35% in 45 min were used. PRM analysis was performed on a Q Exactive HF mass spectrometer (Thermo Scientific, USA). Methods optimized for collision energy, charge state, and retention times on the most significantly regulated peptides were generated experimentally using unique peptides of high intensity and confidence for each target protein. The working parameters of the mass spectrometer were shown in Supplementary material 1. The raw data were analyzed using Skyline (MacCoss Lab, University of Washington), where signal intensities for individual peptide sequences for each of the significantly altered proteins were quantified relative to each sample and normalized to a standard reference.

## Results

### Identification of differentially expressed proteins in calves with diarrhea

**Figure 1:**
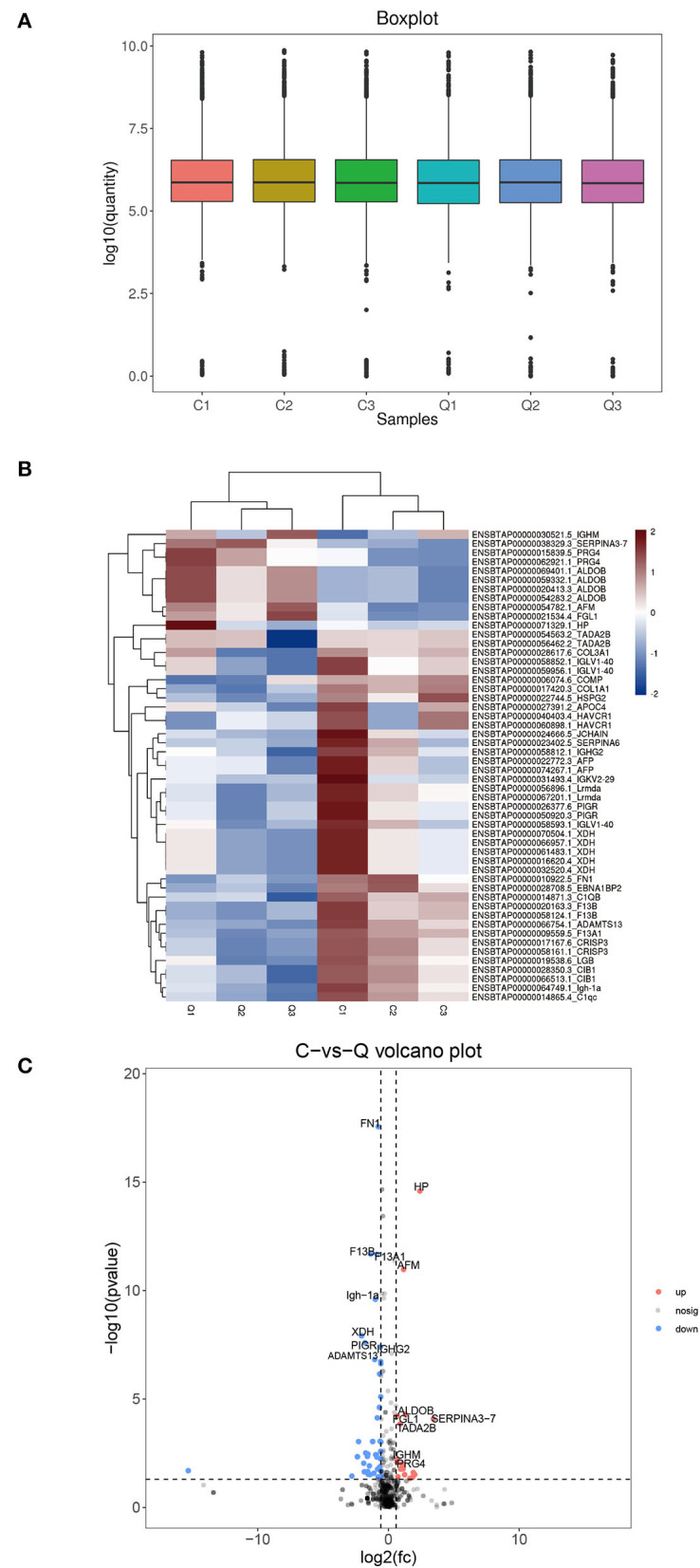
The differential plasma proteins in DHD calves and clinically healthy calves. C indicates clinically healthy calves and Q indicates DHD calves. **(A)** Normalized box diagram. The distribution of the normalized quantitative value of the peptide showed that the signal intensity of each group was basically the same. **(B)** Heatmap of the differentially expressed proteins. Plasma samples represent in the columns, and the differentially expressed proteins are delineated in rows. The color of each cell shows that red represents upregulation and blue represents downregulation. **(C)** Volcano plot of proteins with the most significantly different abundance levels in plasma samples of calves with DHD. Increased protein levels are presented on the positive X-axis and decreased levels are presented on the negative X-axis.

As shown in Figure 1A, the box plot analysis showed that the log10 protein intensity medians of all pooled samples were nearly at the same levels, which suggested that there was no bias toward samples. We used the local normalization method in Pulsar software to normalize the peak intensity of the overall sample spectra. After normalization, a total of 679 proteins were identified in the test group, in that 52 proteins were significantly different between healthy and DHD calves (|FC| > 1.5, *P* < 0.05; Table 4), and 13 proteins of them were upregulated and 39 proteins of them were downregulated in calves with DHD compared with clinically healthy calves (Figure 1B). The specific information of these significantly different proteins, such as gene names, fold change, and annotation information were listed in Table 2. Volcano plots revealed that haptoglobin precursor (HP) and endopin 2C-like (SERPINA3–7) were significantly increased (the right part of the axis), and xanthine dehydrogenase/oxidase (XDH), heparan sulfate proteoglycan 2 (HSPG2), leucine-rich repeat-containing protein C10orf11 homolog (Lrmda), and polymeric immunoglobulin receptor (PIGR) were significantly decreased (the left part of the axis; Figure 1C).

**Table 4:** List of significantly regulated proteins in plasm of DHD calves vs. healthy caves.

**Protein ID**	**Symbol**	**Description**	***P*** **-value**	**log_2_fc**	**Regulation**
ENSBTAP00000038329.3	SERPINA3–7	TPA: Endopin 2C-like [*Bos taurus*]	0.000	3.393	Up
ENSBTAP00000071329.1	HP	haptoglobin precursor [*Bos taurus*]	0.000	2.354	Up
ENSBTAP00000020413.3	ALDOB	ALDOB protein [*Bos taurus*]	0.000	1.246	Up
ENSBTAP00000054283.2	ALDOB	Fructose-bisphosphate aldolase B, partial [*Bos mutus*]	0.000	1.246	Up
ENSBTAP00000059332.1	ALDOB	ALDOB protein [*Bos taurus*]	0.000	1.246	Up
ENSBTAP00000069401.1	ALDOB	ALDOB protein [*Bos taurus*]	0.000	1.246	Up
ENSBTAP00000054782.1	AFM	Afamin precursor [*Bos taurus*]	0.000	1.114	Up
ENSBTAP00000054563.2	TADA2B	TPA: Ada2b-like [*Bos taurus*]	0.000	0.861	Up
ENSBTAP00000056462.2	TADA2B	Transcriptional adapter 2-beta [*Bos taurus*]	0.000	0.861	Up
ENSBTAP00000030521.5	IGHM	Immunoglobulin M heavy chain secretory form [*Bos taurus*]	0.006	0.648	Up
ENSBTAP00000021534.4	FGL1	PREDICTED: fibrinogen-like protein 1 isoform X1 [*Bos taurus*]	0.000	0.631	Up
ENSBTAP00000015839.5	PRG4	Proteoglycan 4 precursor [*Bos taurus*]	0.008	0.614	Up
ENSBTAP00000062921.1	PRG4	TPA: Proteoglycan 4 [*Bos taurus*]	0.008	0.614	Up
ENSBTAP00000028617.6	COL3A1	Collagen alpha-1(III) chain precursor [*Bos taurus*]	0.001	−0.623	Down
ENSBTAP00000014865.4	C1qc	Complement C1q subcomponent subunit C precursor [*Bos taurus*]	0.000	−0.628	Down
ENSBTAP00000014871.3	C1QB	complement C1q subcomponent subunit B precursor [*Bos taurus*]	0.000	−0.635	Down
ENSBTAP00000006074.6	COMP	RecName: Full = Cartilage oligomeric matrix protein; Short = COMP; Flags: Precursor	0.000	−0.635	Down
ENSBTAP00000040403.4	HAVCR1	Hepatitis A virus cellular receptor 1 precursor [*Bos taurus*]	0.003	−0.640	Down
ENSBTAP00000060898.1	HAVCR1	Hepatitis A virus cellular receptor 1 precursor [*Bos taurus*]	0.003	−0.640	Down
ENSBTAP00000058812.1	IGHG2	IgG3 heavy chain constant region, partial [*Bos taurus*]	0.000	−0.658	Down
ENSBTAP00000028708.5	EBNA1BP2	TPA: EBNA1 binding protein 2 [*Bos taurus*]	0.006	−0.667	Down
ENSBTAP00000027391.2	APOC4	Apolipoprotein C-IV precursor [*Bos taurus*]	0.001	−0.683	Down
ENSBTAP00000058852.1	IGLV1–40	PREDICTED: immunoglobulin lambda-like polypeptide 1 isoform X2 [*Bos taurus*]	0.000	−0.712	Down
ENSBTAP00000059956.1	IGLV1–40	PREDICTED: immunoglobulin lambda-like polypeptide 1 isoform X2 [*Bos taurus*]	0.000	−0.712	Down
ENSBTAP00000017167.6	CRISP3	Cysteine-rich secretory protein 3 precursor [*Bos taurus*]	0.000	−0.731	Down
ENSBTAP00000058161.1	CRISP3	Cysteine-rich secretory protein 3 precursor [*Bos taurus*]	0.000	−0.731	Down
ENSBTAP00000010922.5	FN1	Fibronectin precursor [*Bos taurus*]	0.000	−0.829	Down
ENSBTAP00000023402.5	SERPINA6	PREDICTED: corticosteroid-binding globulin [*Bos taurus*]	0.000	−0.898	Down
ENSBTAP00000009559.5	F13A1	coagulation factor XIII A chain [*Bos taurus*]	0.000	−0.919	Down
ENSBTAP00000058593.1	IGLV1–40	PREDICTED: Immunoglobulin lambda-like polypeptide 1 isoform X2 [*Bos taurus*]	0.005	−0.935	Down
ENSBTAP00000024666.5	JCHAIN	Immunoglobulin J chain precursor [*Bos taurus*]	0.004	−0.959	Down
ENSBTAP00000017420.3	COL1A1	Collagen alpha-1(I) chain precursor [*Bos taurus*]	0.004	−1.024	Down
ENSBTAP00000064749.1	Igh-1a	Membrane-bound immunoglobulin gamma1 heavy chain constant region, partial [*Bos taurus*]	0.000	−1.048	Down
ENSBTAP00000066754.1	ADAMTS13	PREDICTED: LOW QUALITY PROTEIN: A disintegrin and metalloproteinase with thrombospondin motifs 13 [*Bison bison bison*]	0.000	−1.093	Down
ENSBTAP00000028350.3	CIB1	Calcium and integrin-binding protein 1 [*Bos taurus*]	0.001	−1.251	Down
ENSBTAP00000066513.1	CIB1	Calcium and integrin-binding protein 1 [*Bos taurus*]	0.001	−1.251	Down
ENSBTAP00000020163.3	F13B	Coagulation factor XIII B chain precursor [*Bos taurus*]	0.000	−1.405	Down
ENSBTAP00000058124.1	F13B	PREDICTED: coagulation factor XIII B chain-like [*Bubalus bubalis*]	0.000	−1.405	Down
ENSBTAP00000031493.4	IGKV2–29	IGK protein [*Bos taurus*]	0.003	−1.623	Down
ENSBTAP00000022772.3	AFP	Alpha-fetoprotein precursor [*Bos taurus*]	0.004	−1.653	Down
ENSBTAP00000074267.1	AFP	Alpha-fetoprotein precursor [*Bos taurus*]	0.004	−1.653	Down
ENSBTAP00000019538.6	LGB	Beta-lactoglobulin precursor [*Bos taurus*]	0.003	−1.789	Down
ENSBTAP00000026377.6	PIGR	PREDICTED: polymeric immunoglobulin receptor [*Bos indicus*]	0.000	−1.841	Down
ENSBTAP00000050920.3	PIGR	PREDICTED: polymeric immunoglobulin receptor [*Bos indicus*]	0.000	−1.841	Down
ENSBTAP00000016620.4	XDH	TPA: xanthine dehydrogenase/oxidase [*Bos taurus*]	0.000	−2.098	Down
ENSBTAP00000032520.4	XDH	TPA: xanthine dehydrogenase/oxidase [*Bos taurus*]	0.000	−2.098	Down
ENSBTAP00000061483.1	XDH	RecName: Full = Xanthine dehydrogenase/oxidase; Includes: RecName: Full = Xanthine dehydrogenase; Short = XD; Includes: RecName: Full = Xanthine oxidase; Short = XO; AltName: Full = Xanthine oxidoreductase; Short = XOR	0.000	−2.098	Down
ENSBTAP00000066957.1	XDH	TPA: xanthine dehydrogenase/oxidase [*Bos taurus*]	0.000	−2.098	Down
ENSBTAP00000070504.1	XDH	RecName: Full = Xanthine dehydrogenase/oxidase; Includes: RecName: Full = Xanthine dehydrogenase; Short = XD; Includes: RecName: Full = Xanthine oxidase; Short = XO; AltName: Full = Xanthine oxidoreductase; Short = XOR	0.000	−2.098	Down
ENSBTAP00000022744.5	HSPG2	TPA: heparan sulfate proteoglycan 2 [*Bos taurus*]	0.001	−2.333	Down
ENSBTAP00000056896.1	Lrmda	PREDICTED: leucine-rich repeat-containing protein C10orf11 homolog [*Equus asinus*]	0.005	−2.420	Down
ENSBTAP00000067201.1	Lrmda	PREDICTED: leucine-rich repeat-containing protein C10orf11 homolog [*Bos indicus*]	0.005	−2.420	Down

Protein ID, ID number of the protein. Symbol, Symbol name of the protein. Description, Functional description of the protein. P-value, P-value for inspection. Log_2_fc, the fold difference was treated by log_2_, and the DHD group was divided by the healthy group. Regulation, Trend of protein change in the DHD group compared with the healthy group.

### Protein functional annotation and enrichment analysis

#### GO annotation of the significantly different proteins

**Figure 2:**
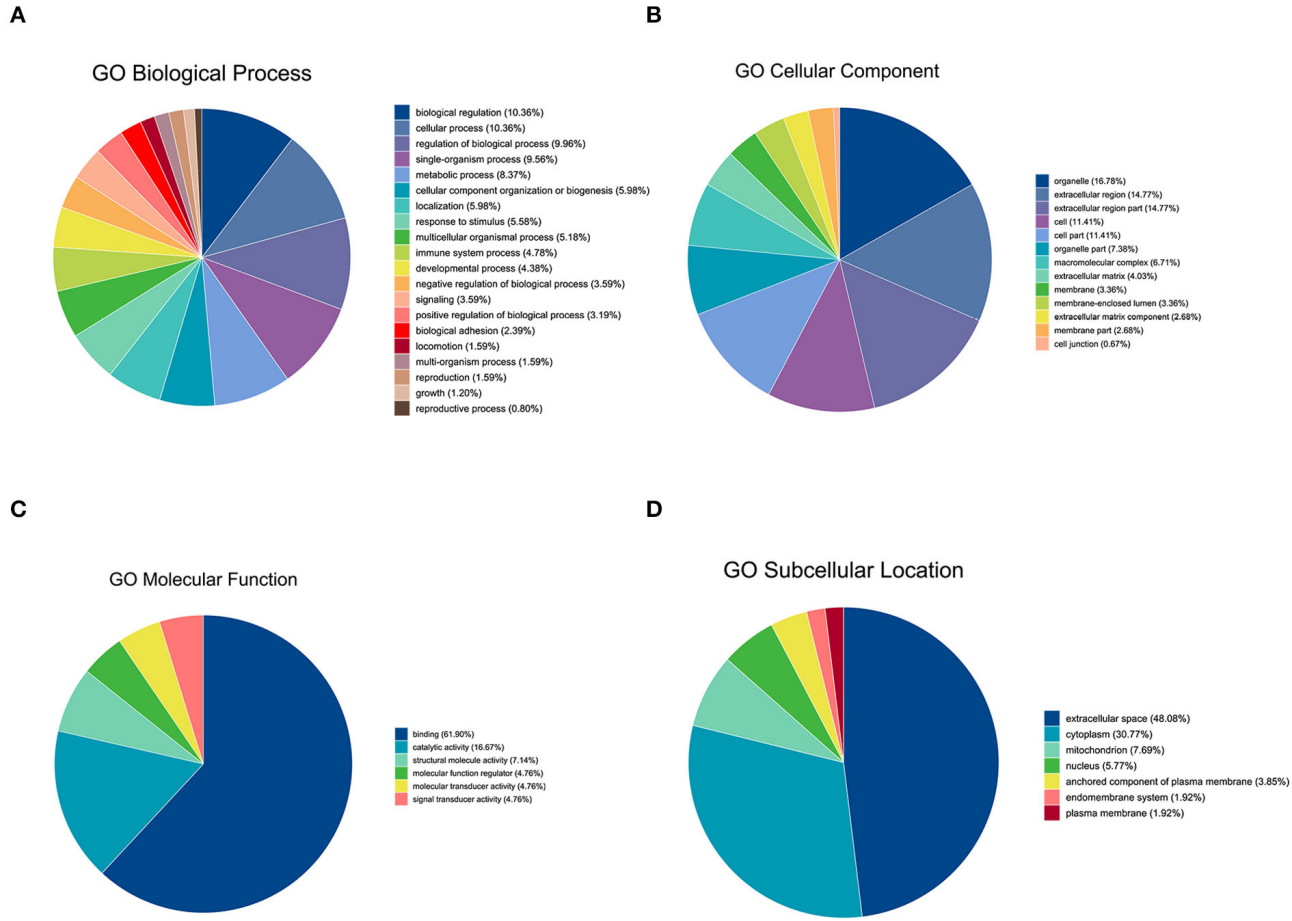
GO analysis of significantly differential proteins between DHD calves and clinically healthy calves. **(A)** Biological process-based analyses of significantly different**ial** proteins. **(B)** Cellular component-based analyses of significantly different**ial** proteins. **(C)** Molecular function-based analyses of significantly different**ial** proteins. **(D)** Subcellular location-based analyses of significantly different expression proteins.

**Figure 3:**
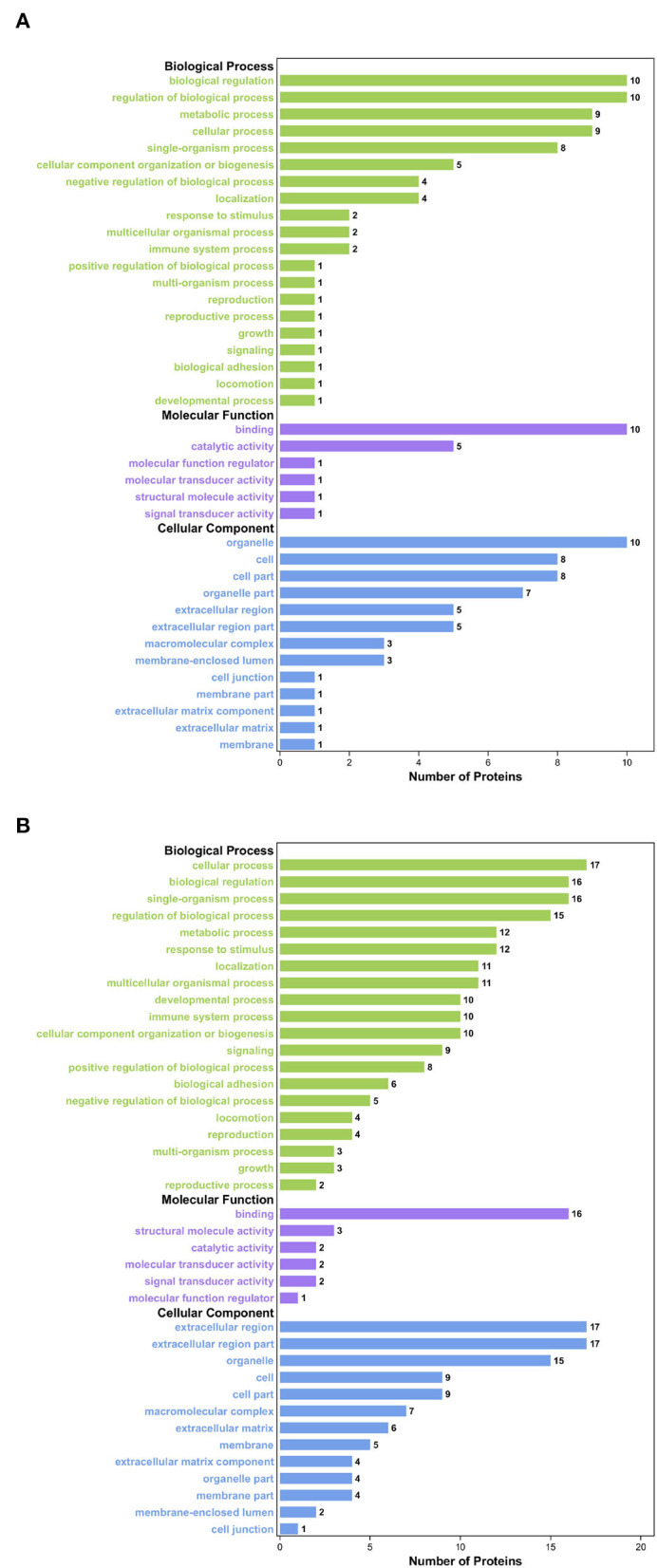
GO enrichment analysis of the significantly differential proteins between DHD calves and clinically healthy calves. **(A)** GO enrichment analysis on the ontology of molecular function, cellular component, and biological process of the significantly increased proteins. **(B)** GO enrichment analysis of the significantly decreased proteins.

Gene Ontology analysis was conducted to explore the ontological functions of 52 differential proteins. Totally, 21 proteins were annotated and classified as participating in certain biological processes, 19 proteins were annotated as related to some molecular function, and the cellular components of 22 proteins were noted. Biological process analysis showed that the majority of the significantly altered proteins were involved in biological regulation (10.36%), cellular process (10.36%), regulation of biological process (9.96%), and single-organism process (9.59%), and the proteins involved in these biological processes included FN1, PIGR, coagulation factor XIII A chain, and complement C1q subcomponent subunit C&B precursor (Figure 2A). Molecular functional analysis demonstrated that the proteins were associated with binding (61.90%), catalytic activity (16.67%), and structural molecule activity (7.14%; Figure 2C). The differential proteins associated with these GO terms include FN1, PIGR, alpha-fetoprotein precursor (AFM), and collagen alpha-1(I) chain precursor (COL1A1). Furthermore, cellular component analysis showed that these significantly altered proteins were located in the organelle (16.78%), extracellular region (14.77%), cell part (11.41%), and organelle part (7.38%; Figure 2B). We also used WoLFPSort to predict the more detailed subcellular localization of these proteins (Figure 2D). They were mainly located in extracellular space (48.08%) and cytoplasm (30.77%), accounting for more than 70% of all significantly different proteins. In addition, these proteins were found in the nucleus, anchored components of the plasma membrane, endomembrane system, and plasma membrane. To further determine which functional categories were closely associated with DHD calves, the significantly increased proteins and significantly decreased proteins were analyzed, respectively, by GO function annotation. For cellular components, the increased proteins were significantly enriched in organelles, whereas decreased proteins were enriched in the extracellular region. The major molecular functions of the increased proteins and the decreased proteins were both bindings. For the biological process, the increased proteins were mainly involved in biological regulation, while decreased proteins participated in the cellular process (Figures 3A,B).

#### KEGG annotation of the significantly different proteins

**Figure 4:**
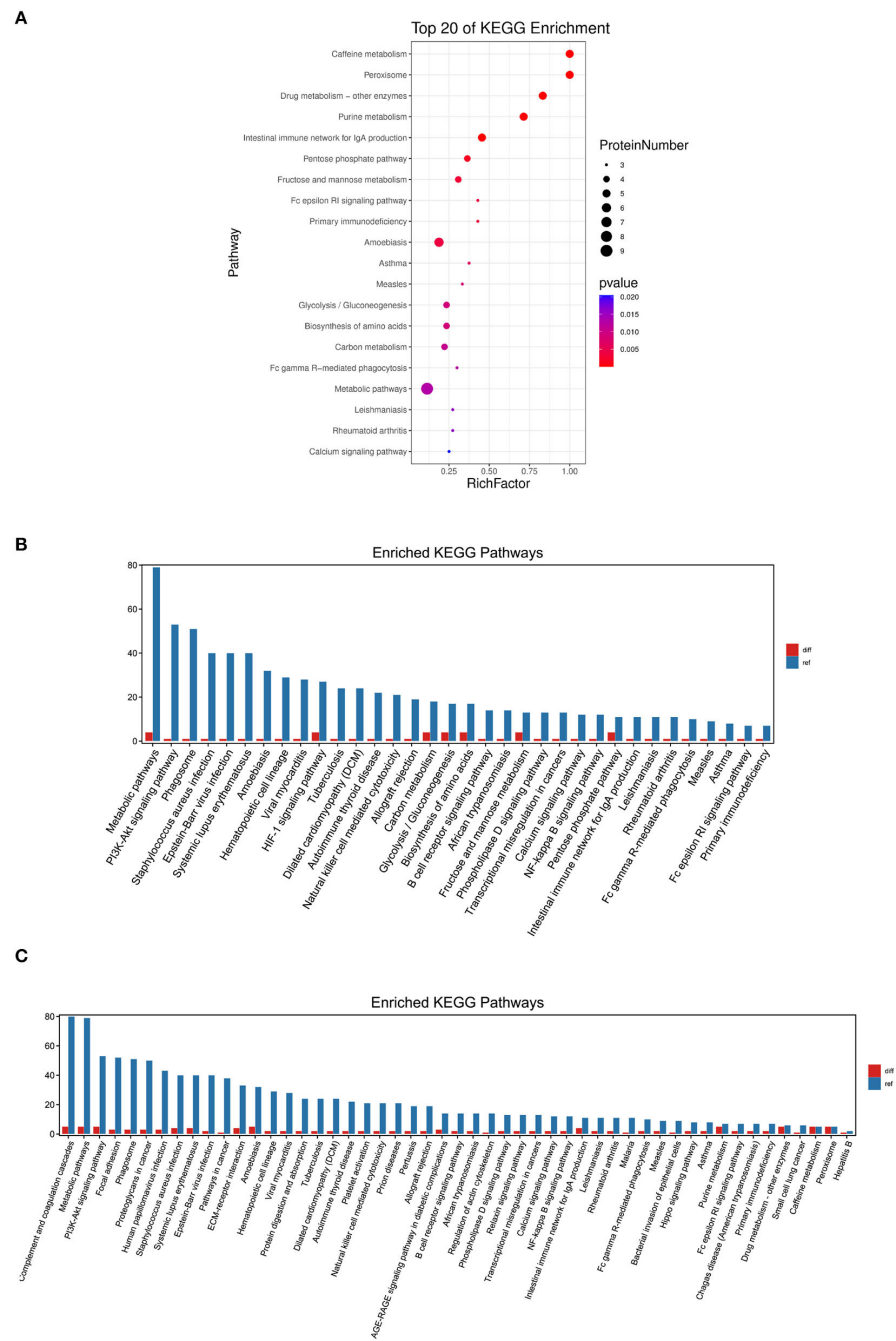
KEGG pathway enrichment analysis of the differentially expressed proteins. **(A)** The first 20 pathways with the lowest *P*-value were used for the diagram, with the ordinate as pathways and the abscissa as enrichment factors (The number of differential proteins in that pathway divided by the number of all proteins. The size indicated the number of different proteins, and the more red the color, the smaller the *P*-value). **(B)** KEGG enrichment analysis of the significantly increased proteins. **(C)** KEGG enrichment analysis of the significantly decreased proteins.

We used KEGG to annotate the information of the significantly different proteins at the level of biological pathways (16). As a result, 57 relevant KEGG signal pathways for 52 differential proteins were annotated, and these proteins were significantly enriched in 31 pathways (*P* < 0.05). The first 20 significantly enriched pathways were shown in Figure 4A, such as caffeine metabolism, intestinal immune network for IgA production, Fc epsilon RI signaling pathway, and NF-κB signaling pathway.

Further comparison of the KEGG pathway enrichment analysis revealed that the increased proteins in calves with DHD were mainly enriched in metabolic pathways, HIF-1 signaling pathway, and fructose and mannose metabolism (Figure 4B), and the decreased proteins were mainly enriched in caffeine metabolism, purine metabolism, and intestinal immune network for IgA production (Figure 4C).

### Network Analysis

**Figure 5:**
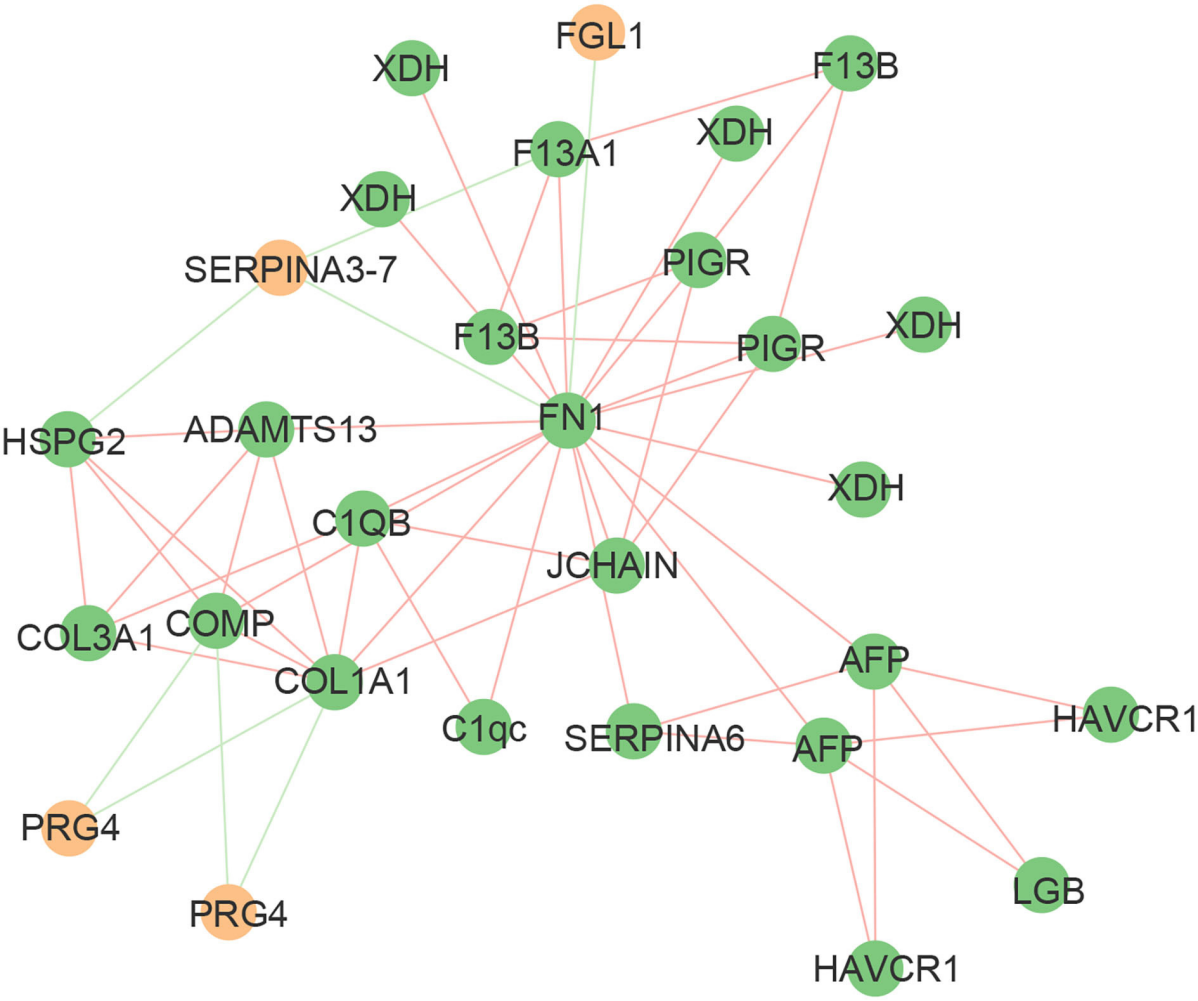
Mapping of significantly differential proteins onto a composite network based on predicted PPI. The edges of the network symbolize different interactions between proteins, based on the actions view of STRING v11.0. The green edges indicate a negative correlation and the red edges indicate a positive correlation. The green nodes indicate upregulated proteins and the yellow nodes indicate downregulated proteins.

The protein-protein interaction (PPI) network consisted of 52 nodes and 113 edges with an average node degree of 8.96 (Figure 5). The expected number of edges for this analysis was 113. FN1, as an important node in the PPI network, was negatively correlated with fibrinogen-like protein 1 isoform X1 (FGL1) and endopin 2C-like (SERPINA3–7), and positively correlated with 15 proteins, such as xanthine dehydrogenase/oxidase (XDH), polymeric immunoglobulin receptor (PIGR), and complement C1q subcomponent subunit C precursor (C1qc). These data indicated that there was a partially biological connection among the differential proteins.

### PRM analysis of the differentially expressed plasma proteins

The results of the PRM pre-experiment showed that 13 of the 52 differential proteins in the test group were detected in the validation group. Thirteen differential proteins corresponding to 38 peptides were selected for PRM validation to verify whether they are associated with DHD. The results of the difference trend and *t*-test showed that fibronectin precursor and apolipoprotein C-IV precursor in plasma of calves with DHD were significantly lower than those in clinically healthy calves (*P* < 0.05), which were consistent with the results from the test group (Table 5). Therefore, the reliability of these two proteins as potential biomarkers for diagnosis of DHD was further validated.

**Table 5:** Differential proteins identified in plasma samples of DHD calves and clinically healthy calves by PRM.

**Protein name**	**Average health**	**Average diarrhea**	**Ratio discovery group (diarrhea/health)**	**Ratio validation group (diarrhea/health)**	***T*** **-Test validation group (diarrhe/health)**
Coagulation factor XIII A chain	0.2071	0.1617	0.528944	0.7808	0.1432
Fibronectin precursor	5.8009	3.6124	0.562785	0.6227	0.0021
Complement C1q subcomponent subunit C precursor	1.3160	1.1665	0.64712	0.8864	0.5312
Complement C1q subcomponent subunit B precursor	0.9890	0.8203	0.644032	0.8294	0.1728
Fibrinogen-like protein 1 isoform X1	0.1047	0.0969	1.548264	0.9258	0.7931
Apolipoprotein C-IV precursor	0.1169	0.0337	0.622963	0.2882	0.0004
Immunoglobulin M heavy chain secretory form	1.5678	2.1028	1.566478	1.3412	0.3602
IGK protein	0.1038	0.1001	0.324764	0.9646	0.9166
Endopin 2C-like	1.1420	0.8088	10.50241	0.7083	0.6404
IgG3 heavy chain constant region	4.0449	4.4027	0.63393	1.0885	0.8002
Immunoglobulin lambda-like polypeptide 1 isoform X2	12.8527	16.0433	0.610376	1.2482	0.7057
Hepatitis A virus cellular receptor 1 precursor	1.0260	0.7950	0.641591	0.7749	0.3276
Haptoglobin precursor	0.1093	4.3719	5.11288	39.9925	0.2805

Average health means a relative expression of the target peptide in clinically healthy calves. Average diarrhea means a relative expression of target peptides in DHD calves. Ratio test group (DHD/health) means the ratio of average expression of target protein between DHD calves and clinically healthy calves in the test group. Ratio validation group (DHD/health) means the ratio of average expression of target protein between DHD calves and clinically healthy calves in the validation group. T-test (P-values) validation group (DHD/health), P-values of t-tests on differential expression of target proteins between DHD calves and clinically healthy calves in the validation group.

## Discussion

Calf diarrhea is a growing concern worldwide because it can cause serious economic losses. DHD is a common type of diarrhea in calves, but the exact pathogenesis has not been determined yet, so the current treatment strategy cannot satisfactorily control the occurrence of DHD in calves. Therefore, it is necessary to further study the pathogenesis of DHD in calves. Proteomics is an effective technique to help characterize diseases by identifying subtle changes in protein abundance between the sick and healthy states of the body (17). To date, although the proteomic has been widely used in the study of many diseases including diarrhea, little information is available about the protein profiles of DHD in the calf. In the present study, proteomics was used to identify significantly differential proteins in calves with DHD, and their possible related functions were analyzed to elucidate the pathogenesis of DHD in calves at the proteomic level.

Intestinal tissue from animals cannot easily be sampled in general, especially in calves, the individual economic value is high. In contrast, the plasma is an ideal sample for studying the pathogenesis of calves with DHD because of its relative chemical and physical stability, abundant protein content, and relatively reliable collection methods in large animals. In this study, 52 proteins were detected to be significantly altered in calves with DHD. KEGG annotated that these proteins are involved in caffeine metabolism, Fc epsilon RI signal pathway, purine metabolism, intestinal immune network for IgA production, NF-κB signal pathway, and PI3K/Akt signal pathway. Caffeine metabolism and purine metabolism are important metabolic processes in the body, and their metabolic disorder will lead to the occurrence of many diseases, such as rheumatic immune diseases and obesity (18, 19). Caffeine was verified to be closely related to inhibiting diarrhea, the reason is related to its function of sterilization and anti-inflammatory effect (20, 21). Purines are natural substances found in virtually all foods, and they can be metabolized into uric acid in the body, which has antioxidant properties (22). Besides, purines as chemical messengers could transmit throughout tissues and species, and cross-linked with other transmitter networks to coordinate numerous aspects of cell behavior such as proliferation, migration, apoptosis, and other physiological processes of organisms (18). It has also been shown that regulation of purine metabolism can effectively alleviate colitis (23). Fc epsilon RI signal pathway plays a central role in the IgE-mediated allergic response and mast cell inflammation, which could be activated in some diseases with increased inflammation such as digestive diseases and heart diseases (24, 25). The significant increase in IGHM associated with this pathway in calves with DHD may indicate an enhanced inflammatory response in sick calves. PI3K/Akt signal pathway participates in the pathogenesis of diarrhea-related diseases by regulating various inflammatory factors (26). The NF-κB transcription factor is a typical pro-inflammatory signaling factor, and the activation of NF-κB can promote the occurrence and development of downstream inflammatory reactions (27). Abnormal regulation of these pathways has been found in several diseases presenting with diarrhea (28, 29).

Most of the significantly differential proteins (39/52) in this study were decreased in calves with DHD, including fibronectin, coagulation factor XIII B chain, and coagulation factor XIII A chain. Fibronectin is a glycoprotein that is involved in various biochemical processes, such as wound healing, cell adhesion, and blood coagulation (30, 31). Fibronectin decreased in calves with DHD, which may be responsible for the increased mucosal permeability (32). Adhesion is a crucial step in bacterial infection. Fibronectin was found to be associated with adhesion in many intestinal pathogens (33). Previous studies have considered fibronectin as a biomarker for some diseases, such as sepsis, where low fibronectin levels seem to be a marker of poor prognosis, and meningitis and asthma, where fibronectin levels are significantly increased (34, 35). But it is the first time that fibronectin could be a biomarker for calf diarrhea. Coagulation factor XIII is a coagulation factor with many cellular functions, which is involved in the wound healing process, proangiogenic function, and monocyte/macrophage functions (36). It has been reported that the levels and activity of coagulation factor XIII antigen were reduced in inflammatory bowel disease (37). Plasma coagulation factor XIII could promote cross-linking of the extracellular matrix components fibrin and fibronectin in response to tissue injury (38). The cross-linking of coagulation factor XIII A to fibronectin could promote the wound healing process (39). In this study, the reduction of these proteins may slow down the healing process of intestinal injury in calves with DHD. Heparan sulfate proteoglycans, complex molecules in cell membrane and extracellular matrix, play vital roles in tumorigenesis due to its mediating cell adhesion, differentiation, migration, and signal transduction. It also has been considered as an important target for the treatment and diagnosis of colorectal cancer (40). Xanthine dehydrogenase (XDH) and Xanthine oxidase (XO) are two interconvertible forms of Xanthine oxidoreductase (XOR). XO activity could be inhibited in some diseases that can lead to intestinal injury (41). Metabolism of intestinal tissue is usually very active, even short periods of hypoxia or ischemia may cause oxidative damage to the intestinal mucosa, which is due to an increase in the amount or activity of XO. It has been demonstrated that XDH is converted to XO when intestinal mucosa was oxidative damaged (42). Therefore, this may also be one of the reasons for the significant decrease in XDH in calves with DHD. In addition, alpha-fetoprotein (AFP) (43), IGK protein (44), and collagen alpha-1 (I) chain precursor (COL1A1) (45) were also used as biomarkers for some intestinal diseases.

The significantly increased proteins of plasma in calves with DHD included haptoglobin precursor, afamin precursor, and ALDOB protein. Haptoglobin, an acute phase response protein secreted by the liver, has the activities of antioxidant, immunomodulatory, antibacterial, and anti-inflammatory (46, 47). The aberrant glycosylation of haptoglobin is associated with many diseases, especially cancer (48). In addition, as a biomarker of diabetic cardiovascular disease and steroid-resistant nephrotic syndrome, haptoglobin can also be used to evaluate and diagnose diarrhea in calves due to its significantly increased expression in calves with DHD (49–51). The significantly increased haptoglobin in the plasma of calves with DHD may be associated with an acute inflammatory response caused by diarrhea. Afamin is a vitamin E-binding glycoprotein expressed mainly in the liver and is associated with many metabolic diseases, such as type 2 diabetes, metabolic syndrome, and obesity (52–54). Afamin is also considered to be a marker of increased hepatic lipid content, and fatty liver has been found in damp-heat diarrhea in rats (52, 55). Although this protein has not been reported in diarrhea-related diseases, it is one of the biomarkers for the detection of gastric cancer (56), and it plays an important role in the anti-apoptotic cellular processes related to oxidative stress, which may be the reason for the significantly increased afamin precursor in the plasma of calves with DHD (57). ALDOB played an important role in glycolysis and was annotated by metabolic pathways, carbon metabolism, glycolysis/gluconeogenesis, and HIF-1 signaling pathway by KEGG pathway analysis. ALDOB is mainly used for the diagnosis of liver diseases and is used as a biomarker for acute liver injury (58). ALDOB has also been poorly studied in relation to diarrhea, but the plasma glucose and lactate concentrations could change in calves with DHD (59), which could account for the significant changes in ALDOB protein. In addition, proteoglycan 4 is a protein that is critical for the virulence factor binding to the cell surface, so it plays an important role in colitis (60).

In the biomarker validation group, the reliability of FN1 and APOC4 as biomarkers for a calf with DHD was verified by targeted quantification. FN1 is related to tissue repair and host defense, and it is also annotated by KEGG into the PI3K-Akt signaling pathway, focal adhesion, and bacterial invasion of epithelial cells. Apolipoprotein could be synthesized in the intestine and reduced inflammatory responses (61). A significant reduction of Apolipoprotein has also been reported in some cases of diarrhea, which may be related to its important role in lipid metabolism in intestinal diseases (62, 63). Therefore, these two proteins may be potential biomarkers for diagnosis and targets for therapy on DHD in calves. In addition, although there were no significant differences in coagulation factor XIII A chain, IGK protein, and haptoglobin precursor in the invalidation group, their changing trends were consistent with those in the test group. These proteins may be closely associated with the pathogenesis of DHD in calves.

This is a preliminary study that indicates the feasibility of plasma proteins as a potential diagnostic biomarker approach for calves with DHD. Since our experiments were conducted on one farm, there are significant limitations in the results and conclusion. In our follow-up experiments, the number of clinical samples and farms should be increased, and more testing methods, such as enzyme-linked immunosorbent assay (ELISA) and western blot, are needed to verify these potential biomarkers for the clinical diagnosis and reliable targets for therapy DHD in calves. In addition, a more in-depth analysis of plasma proteomics in combination with other omics can provide a more complete and accurate understanding of calves with DHD.

## Conclusion

Dampness heat diarrhea disturbed the composition of plasma proteins in calves. The changes in plasma protein levels, especially fibronectin precursor, haptoglobin precursor, coagulation factor XIII A chain, and apolipoprotein C-IV precursor, might affect the progression of DHD in calves by interfering with complement and coagulation cascades, PI3K-Akt signaling pathway, and focal adhesion. The reliability of fibronectin precursor and apolipoprotein C-IV precursor as biomarkers of calf with DHD was verified using PRM analysis. The findings provide a new insight for further exploring the mechanism of DHD in calves and evaluating the clinical meaning of fibronectin precursor and apolipoprotein C-IV precursor for diagnosis or treatment of the disease.

## Data availability statement

The datasets presented in this study can be found in online repositories. The names of the repository/repositories and accession number(s) can be found at: http://www.proteomexchange.org/, PXD027099.

## Ethics statement

The animal study was reviewed and approved by Laboratory Animal Ethics Commission of the Lanzhou Institute of Husbandry and Pharmaceutical Sciences of CAAS.

## Author contributions

JL and KaiZ conceived and designed the work. JL coordinated technical support and funding. ZY wrote the manuscript. ZY, KanZ, KaiZ, GW, LW, JZ, ZQ, and ZG performed the experiments and collected the samples. JL and KaiZ reviewed the manuscript. All authors have read and approved the published version of the manuscript.

## Funding

This work was financially supported by the Earmarked Fund for CARS-36, the National Key Research and Development Program of China (2017YFE0114400 and 2018YFD0502403), and Special Fund of Chinese Central Government for Basic Scientific Research Operations in Commonwealth Research Institutes (1610032021002).

## Conflict of interest

The authors declare that the research was conducted in the absence of any commercial or financial relationships that could be construed as a potential conflict of interest.

## Publisher's note

All claims expressed in this article are solely those of the authors and do not necessarily represent those of their affiliated organizations, or those of the publisher, the editors and the reviewers. Any product that may be evaluated in this article, or claim that may be made by its manufacturer, is not guaranteed or endorsed by the publisher.
