# Supplementary material for: Differential proteomic of plasma provides a new perspective on scientific diagnosis and drug screening for dampness heat diarrhea in calves
Source: Front Vet Sci. 2022 Sep 20;9:986329. doi: 10.3389/fvets.2022.986329 (PMC9530945; doi:10.3389/fvets.2022.986329)
Supplement: Supplementary file 1 [file Data_Sheet_1.doc]

**DDA: nano-HPLC-MS/MS Analysis**

The peptides were re-dissolved in 30 μL solvent A (A: 0.1% formic acid in water) and analyzed by on-line nanospray LC-MS/MS on an Orbitrap Fusion Lumos coupled to EASY-nLC 1200 system (Thermo Fisher Scientific, MA, USA). 3 μL peptide sample was loaded onto the analytical column (Acclaim PepMap C18, 75 μm x 25 cm) and separated with a 120-min gradient, from 5% to 35% B (B: 0.1% formic acid in ACN). The column flow rate was maintained at 200 nL/min with the column temperature of 40°C. The electrospray voltage of 2 kV versus the inlet of the mass spectrometer was used.

The mass spectrometer was run under data dependent acquisition mode, and automatically switched between MS and MS/MS mode. The parameters was: (1) MS: scan range (m/z) =350–1200; resolution=120,000; AGC target=400000; maximum injection time=50 ms; Filter Dynamic Exclusion: exclusion duration=30s; (2) HCD-MS/MS: resolution=15,000; AGC target=50000; maximum injection time=35 ms; collision energy=32.
