# Supplementary material for: Differential proteomic of plasma provides a new perspective on scientific diagnosis and drug screening for dampness heat diarrhea in calves
Source: Front Vet Sci. 2022 Sep 20;9:986329. doi: 10.3389/fvets.2022.986329 (PMC9530945; doi:10.3389/fvets.2022.986329)
Supplement: Supplementary file 2 [file Data_Sheet_2.docx]

**Table S1** the clinical manifestations of DHD calves

| Ear label of Case | Clinical signs | | | | | | |
| --- | --- | --- | --- | --- | --- | --- | --- |
|  | diarrhea | mucus or bloody or purulent stool | red tongue and thick greasy tongue-coating | hyperthermia, dry nose | abdominal pain, tenesmus | anal red and swell | thirst and small amount, shortness of urination |
| 201247 | ＋ | ＋ | ＋ | ＋ | － | ＋ | － |
| 201273 | ＋ | ＋ | ＋ | ＋ | － | ＋ | － |
| 201270 | ＋ | ＋ | ＋ | ＋ | － | ＋ | ＋ |
| 201272 | ＋ | ＋ | ＋ | ＋ | － | ＋ | － |
| 201253 | ＋ | ＋ | ＋ | ＋ | － | ＋ | － |
| 201259 | ＋ | ＋ | ＋ | ＋ | － | ＋ | － |
| 201268 | ＋ | ＋ | － | ＋ | － | ＋ | － |
| 201193 | ＋ | ＋ | － | ＋ | － | ＋ | － |
| 201183 | ＋ | ＋ | － | ＋ | － | ＋ | － |
| 201195 | ＋ | ＋ | ＋ | ＋ | － | ＋ | － |
| 201106 | ＋ | ＋ | ＋ | ＋ | ＋ | ＋ | ＋ |
| 201117 | ＋ | ＋ | － | ＋ | ＋ | ＋ | － |
| 201138 | ＋ | ＋ | ＋ | ＋ | ＋ | － | － |
| 201118 | ＋ | ＋ | ＋ | ＋ | － | ＋ | － |
| 201197 | ＋ | ＋ | ＋ | ＋ | － | ＋ | － |
| 201139 | ＋ | － | ＋ | ＋ | － | ＋ | － |
| 201103 | ＋ | ＋ | － | ＋ | ＋ | － | ＋ |
| 201105 | ＋ | ＋ | － | ＋ | ＋ | － | － |
| 201170 | ＋ | ＋ | － | ＋ | ＋ | ＋ | ＋ |
| 201188 | ＋ | ＋ | ＋ | － | － | ＋ | ＋ |
| 201158 | ＋ | ＋ | ＋ | － | － | ＋ | － |
| 201163 | ＋ | ＋ | ＋ | ＋ | － | ＋ | － |
| 201162 | ＋ | ＋ | ＋ | ＋ | － | ＋ | － |
| 201190 | ＋ | ＋ | ＋ | ＋ | － | ＋ | － |
| 201290 | ＋ | ＋ | － | － | － | ＋ | － |
| 201218 | ＋ | ＋ | － | ＋ | ＋ | ＋ | － |
| 201219 | ＋ | ＋ | － | ＋ | － | ＋ | － |
| 201255 | ＋ | ＋ | － | － | － | ＋ | ＋ |
| 201232 | ＋ | ＋ | － | ＋ | － | ＋ | － |
| 201261 | ＋ | ＋ | ＋ | ＋ | ＋ | ＋ | － |
| 201265 | ＋ | ＋ | ＋ | ＋ | ＋ | ＋ | － |
| 201266 | ＋ | ＋ | ＋ | ＋ | ＋ | ＋ | － |
| 201267 | ＋ | ＋ | － | ＋ | － | ＋ | － |
| 201279 | ＋ | ＋ | － | ＋ | － | ＋ | － |
| 201277 | ＋ | ＋ | － | ＋ | － | ＋ | － |
| 201281 | ＋ | ＋ | － | ＋ | ＋ | ＋ | ＋ |
| 201282 | ＋ | ＋ | － | ＋ | ＋ | ＋ | － |
| 201287 | ＋ | ＋ | － | ＋ | ＋ | － | － |
| 201291 | ＋ | ＋ | － | ＋ | ＋ | ＋ | － |
| 201292 | ＋ | ＋ | ＋ | ＋ | － | ＋ | － |
| 201299 | ＋ | ＋ | ＋ | － | － | ＋ | － |
| 201301 | ＋ | ＋ | ＋ | ＋ | ＋ | － | － |
| 201306 | ＋ | ＋ | ＋ | ＋ | ＋ | － | － |
| 201307 | ＋ | ＋ | ＋ | － | ＋ | － | － |
| 201309 | ＋ | － | ＋ | － | ＋ | ＋ | ＋ |
| 201312 | ＋ | － | ＋ | － | ＋ | ＋ | ＋ |
| 201313 | ＋ | ＋ | ＋ | ＋ | － | ＋ | － |
| 201319 | ＋ | ＋ | ＋ | ＋ | ＋ | ＋ | ＋ |
| 201326 | ＋ | ＋ | － | ＋ | ＋ | ＋ | － |
| 201327 | ＋ | ＋ | － | ＋ | ＋ | － | － |
| 201334 | ＋ | ＋ | ＋ | ＋ | ＋ | － | － |
| 201346 | ＋ | ＋ | ＋ | ＋ | ＋ | ＋ | － |
| 201357 | ＋ | ＋ | ＋ | ＋ | ＋ | ＋ | － |
| 201358 | ＋ | ＋ | ＋ | ＋ | － | ＋ | － |
| 201364 | ＋ | ＋ | ＋ | ＋ | － | ＋ | － |
| 201369 | ＋ | ＋ | ＋ | ＋ | － | ＋ | － |
| 201371 | ＋ | ＋ | ＋ | ＋ | ＋ | ＋ | － |
| 201372 | ＋ | ＋ | ＋ | － | ＋ | ＋ | － |
| 201373 | ＋ | ＋ | ＋ | ＋ | ＋ | ＋ | － |
| 201385 | ＋ | ＋ | ＋ | ＋ | ＋ | ＋ | － |
| 201386 | ＋ | － | ＋ | ＋ | － | ＋ | － |
| 201391 | ＋ | ＋ | ＋ | ＋ | ＋ | ＋ | ＋ |
| 201401 | ＋ | ＋ | ＋ | ＋ | ＋ | ＋ | － |
| 201403 | ＋ | ＋ | ＋ | － | ＋ | ＋ | － |
| 201406 | ＋ | ＋ | ＋ | ＋ | ＋ | － | － |
| 201410 | ＋ | ＋ | ＋ | ＋ | ＋ | ＋ | － |
| 201411 | ＋ | ＋ | ＋ | ＋ | ＋ | ＋ | － |
| 201415 | ＋ | ＋ | ＋ | ＋ | ＋ | ＋ | ＋ |
| 201416 | ＋ | ＋ | ＋ | ＋ | ＋ | ＋ | － |
| 201420 | ＋ | ＋ | ＋ | ＋ | ＋ | ＋ | － |
| 201421 | ＋ | ＋ | ＋ | ＋ | ＋ | ＋ | － |
| 201425 | ＋ | ＋ | ＋ | ＋ | ＋ | ＋ | － |
| 201426 | ＋ | ＋ | ＋ | ＋ | － | ＋ | － |
| 201428 | ＋ | ＋ | ＋ | ＋ | ＋ | ＋ | － |
| 201435 | ＋ | ＋ | ＋ | ＋ | ＋ | ＋ | － |
| 201436 | ＋ | － | ＋ | ＋ | ＋ | ＋ | － |
| 201444 | ＋ | ＋ | － | － | ＋ | ＋ | － |
| 201446 | ＋ | ＋ | － | ＋ | ＋ | ＋ | － |
| 201457 | ＋ | ＋ | ＋ | ＋ | ＋ | ＋ | ＋ |
| 201458 | ＋ | ＋ | ＋ | ＋ | ＋ | ＋ | － |
| 201461 | ＋ | － | ＋ | ＋ | ＋ | － | ＋ |
| 201468 | ＋ | ＋ | ＋ | ＋ | ＋ | ＋ | － |
| 201472 | ＋ | ＋ | ＋ | ＋ | ＋ | ＋ | － |
| 201473 | ＋ | ＋ | － | ＋ | ＋ | ＋ | － |
| 201475 | ＋ | ＋ | ＋ | ＋ | ＋ | ＋ | － |
| 201483 | ＋ | ＋ | － | ＋ | ＋ | － | ＋ |
| 201484 | ＋ | ＋ | ＋ | ＋ | ＋ | ＋ | － |
| 201485 | ＋ | ＋ | ＋ | ＋ | ＋ | － | － |
| 201486 | ＋ | ＋ | ＋ | ＋ | ＋ | ＋ | － |
| 201491 | ＋ | ＋ | ＋ | ＋ | ＋ | ＋ | － |
| 201493 | ＋ | ＋ | ＋ | ＋ | ＋ | ＋ | － |
| 201495 | ＋ | － | ＋ | ＋ | ＋ | ＋ | － |
| 201501 | ＋ | ＋ | ＋ | ＋ | － | ＋ | － |
| 201503 | ＋ | ＋ | － | ＋ | － | ＋ | ＋ |
| 201504 | ＋ | ＋ | － | ＋ | － | ＋ | － |
| 201511 | ＋ | ＋ | － | ＋ | ＋ | ＋ | ＋ |
| 201512 | ＋ | ＋ | ＋ | ＋ | ＋ | ＋ | ＋ |
| 201513 | ＋ | ＋ | ＋ | ＋ | ＋ | ＋ | ＋ |
| 201517 | ＋ | ＋ | ＋ | ＋ | － | ＋ | － |
| 201525 | ＋ | ＋ | ＋ | ＋ | ＋ | ＋ | － |
| 201526 | ＋ | － | ＋ | ＋ | ＋ | ＋ | － |
| 201527 | ＋ | ＋ | ＋ | ＋ | ＋ | ＋ | － |
| 201528 | ＋ | ＋ | ＋ | ＋ | ＋ | ＋ | － |
| 201532 | ＋ | ＋ | ＋ | ＋ | ＋ | ＋ | － |
| 201534 | ＋ | ＋ | ＋ | ＋ | ＋ | ＋ | － |
| 201535 | ＋ | ＋ | ＋ | ＋ | ＋ | － | － |
| 201538 | ＋ | ＋ | ＋ | ＋ | ＋ | ＋ | － |
| 201539 | ＋ | ＋ | ＋ | － | ＋ | ＋ | － |
| 201541 | ＋ | ＋ | ＋ | ＋ | － | ＋ | － |
| 201545 | ＋ | ＋ | ＋ | － | ＋ | ＋ | － |
| 201547 | ＋ | ＋ | ＋ | ＋ | ＋ | ＋ | － |
| 201549 | ＋ | ＋ | ＋ | ＋ | ＋ | ＋ | ＋ |
| 201550 | ＋ | ＋ | ＋ | ＋ | ＋ | ＋ | ＋ |
| 201551 | ＋ | ＋ | ＋ | ＋ | ＋ | ＋ | － |
| 201552 | ＋ | ＋ | ＋ | ＋ | ＋ | － | － |
| 201556 | ＋ | ＋ | ＋ | ＋ | ＋ | － | － |
| 201558 | ＋ | ＋ | ＋ | － | ＋ | ＋ | － |
| 201559 | ＋ | ＋ | ＋ | ＋ | ＋ | － | － |
| 201560 | ＋ | ＋ | ＋ | ＋ | ＋ | ＋ | － |
| 501564 | ＋ | － | ＋ | ＋ | － | ＋ | － |

Note: ＋ indicates that this symptom was observed. －indicates that this symptom was not observed.

**Table S2** the clinical manifestations of clinically healthy calves

| Ear label of Case | Clinical signs | | | |  |
| --- | --- | --- | --- | --- | --- |
|  | Mobility | Body temperature | Stool form | appetite | age |
| 201098 | lively and active | normal | formed stool | good | 14-day-old |
| 201099 | lively and active | normal | formed stool | good | 13-day-old |
| 201104 | lively and active | normal | formed stool | good | 13-day-old |
| 201107 | lively and active | normal | formed stool | good | 16-day-old |
| 201108 | lively and active | normal | formed stool | good | 13-day-old |
| 201109 | lively and active | normal | formed stool | good | 10-day-old |
| 201110 | lively and active | normal | formed stool | good | 10-day-old |
| 201112 | lively and active | normal | formed stool | good | 15-day-old |
| 201113 | lively and active | normal | formed stool | good | 12-day-old |
| 201115 | lively and active | normal | formed stool | good | 8-day-old |
| 201119 | lively and active | normal | formed stool | good | 15-day-old |
| 201120 | lively and active | normal | formed stool | good | 13-day-old |
| 201121 | lively and active | normal | formed stool | good | 13-day-old |
| 201122 | lively and active | normal | formed stool | good | 12-day-old |
| 201123 | lively and active | normal | formed stool | good | 10-day-old |
| 201125 | lively and active | normal | formed stool | good | 15-day-old |
| 201126 | lively and active | normal | formed stool | good | 14-day-old |
| 201127 | lively and active | normal | formed stool | good | 11-day-old |
| 201130 | lively and active | normal | formed stool | good | 11-day-old |
| 201140 | lively and active | normal | formed stool | good | 10-day-old |
| 201141 | lively and active | normal | formed stool | good | 17-day-old |
| 201142 | lively and active | normal | formed stool | good | 10-day-old |
| 201146 | lively and active | normal | formed stool | good | 12-day-old |
| 201151 | lively and active | normal | formed stool | good | 15-day-old |
| 201152 | lively and active | normal | formed stool | good | 14-day-old |
| 201153 | lively and active | normal | formed stool | good | 8-day-old |
| 201171 | lively and active | normal | formed stool | good | 10-day-old |
| 201172 | lively and active | normal | formed stool | good | 9-day-old |
| 201175 | lively and active | normal | formed stool | good | 9-day-old |
| 201176 | lively and active | normal | formed stool | good | 9-day-old |
| 201201 | lively and active | normal | formed stool | good | 8-day-old |
| 201202 | lively and active | normal | formed stool | good | 11-day-old |
| 201203 | lively and active | normal | formed stool | good | 10-day-old |
| 201205 | lively and active | normal | formed stool | good | 10-day-old |
| 201206 | lively and active | normal | formed stool | good | 9-day-old |
| 201221 | lively and active | normal | formed stool | good | 9-day-old |
| 201222 | lively and active | normal | formed stool | good | 8-day-old |
| 201223 | lively and active | normal | formed stool | good | 13-day-old |
| 201228 | lively and active | normal | formed stool | good | 9-day-old |
| 201229 | lively and active | normal | formed stool | good | 15-day-old |
| 201234 | lively and active | normal | formed stool | good | 10-day-old |
| 201235 | lively and active | normal | formed stool | good | 10-day-old |
| 201236 | lively and active | normal | formed stool | good | 17-day-old |
| 201241 | lively and active | normal | formed stool | good | 10-day-old |
| 201242 | lively and active | normal | formed stool | good | 12-day-old |
| 201245 | lively and active | normal | formed stool | good | 15-day-old |
| 201249 | lively and active | normal | formed stool | good | 14-day-old |
| 201260 | lively and active | normal | formed stool | good | 10-day-old |
| 201302 | lively and active | normal | formed stool | good | 10-day-old |
| 201303 | lively and active | normal | formed stool | normal appetite | 12-day-old |
| 201304 | lively and active | normal | formed stool | normal appetite | 10-day-old |
| 201315 | lively and active | normal | formed stool | normal appetite | 10-day-old |
| 201320 | lively and active | normal | formed stool | normal appetite | 11-day-old |
| 201321 | lively and active | normal | formed stool | normal appetite | 9-day-old |
| 201322 | lively and active | normal | formed stool | normal appetite | 9-day-old |
| 201340 | lively and active | normal | formed stool | normal appetite | 8-day-old |
| 201341 | lively and active | normal | formed stool | normal appetite | 12-day-old |
| 201343 | lively and active | normal | formed stool | normal appetite | 17-day-old |
| 201351 | lively and active | normal | formed stool | normal appetite | 9-day-old |
| 201352 | lively and active | normal | formed stool | normal appetite | 9-day-old |
